# Alveolar Abscess

**Published:** 1882-08

**Authors:** M. S. Dean

**Affiliations:** Chicago


### ALVEOLAR ABSCESS.

M. S. Dean, D.D.S., Chicago.

(Read before the International Medical Congress, London, Aug. 6, 1881.)

Mr. President and Gentlemen:—The causes of alveolar abscess and its pathology have been so minutely and accurately described by several learned authors that it would only be a waste of time for me to dwell upon the etiology of this disease, or upon its history. I will, however, state at the outset that I shall denominate as alveolar abscess no lesion except that which is associated with a tooth, the pulp of which has become devitalized; no matter whether the devitalization be of recent or remote occurrence. In other words, the death of the pulp will be regarded as the remote or indirect cause of the disease. The immediate or exciting causes, then, of alveolar abscess (properly so-called) are, the various matters and their products that find their way from the root canals through the apical foramen. These irritating agents may arise either from the decomposition of the pulp tissues, or, at a later period, of the exudations that may have entered the canal through the apical foramen, or from the matters that may have entered the root canal from external sources; or, indeed, in some cases even, from the passage of liquids or gases through the canaliculi of the dentine and cement of the root. Any of these agencies, acting mechanically or septically upon the tissues surrounding the root, would cause inflammation, and, as a consequence, might terminate in alveolar abscess.

This is a very general statement, indeed, of the causes of the lesion which is made the subject of this paper. To trace its progress from its incipiency to its climax would be, as before intimated, only a waste of time, since its different progressive stages are well known to every dental pathologist.

The pathogeny of this disease being thus condensed within these narrow limits, it remains only for me to proceed to the practical question, its treatment. I shall in this confine myself to a few of the ordinary cases that are of the most frequent occurrence, of which some are regarded by many writers as incurable, and, unfortunately, are treated as such by many practitioners.

Alveolar abscess with fistulous outlets, whether of long standing or recent development, are the least difficult to treat, and are the most speedily brought to a favorable termination. Indeed, the treatment of such cases is exceedingly simple; and if the remedial agent is properly and thoroughly applied, a single operation usually results in a permanent cure. To illustrate the mode of treatment in detail, we will take an example in practice. A fistulous abscess associated with a superior bicuspid having a large cavity on its distal surface—the gums somewhat inflamed and turgid. The first step in the treatment of this case—whether the abscess be acute or chronic—consists in adjusting the rubber dam to this and to the adjacent posterior tooth—if there be such. The decayed parts should then be removed, and a convenient entrance made into the root canal or canals, which should be enlarged, if necessary, with a Gate's drill, or other instruments suitable for that purpose, and thoroughly cleansed. A steel broach should then be passed gently through the apical foramen to remove any obstruction to the passage of liquids. This last operation sometimes requires considerable patience, especially so when the end of the root is somewhat curved. The next process consists in the injection of creosote through the apical foramen into the abscess. This operation should not be discontinued until the whitened orifice of the fistula gives evidence that the remedy has reached this point.

A simple method of accomplishing this, and one that has proved entirely satisfactory to myself and to others, is this: Fill the root-canal with a light pledget of cotton, saturated with creosote; then introduce a ball of pure rubber into the cavity of decay; now, with a few quick and forcible impulsions, with a round-headed instrument upon the soft rubber, the creosote will be forced through the apical foramen and the mouth of the fistula. The injection of the abscess and fistulous tract having been accomplished, the root canal or canals may now be filled tightly with cotton, slightly moistened with creosote, and the cavity of decay sealed up so securely as to prevent the entrance of the fluids of the mouth. If the details of this simple operation are carefully carried out, no additional treatment will be necessary in a great majority of cases to effect a permanent cure.

After the lapse of five or six days, the fistula will be found closed, and the pulp canal should now be completely filled with some durable and impervious material. But before the temporary stopping is removed, the rubber dam should again be applied to the tooth in order to prevent the possibility of any contaminating matter entering the root-canals. This last point is so essential that I wish to strongly emphasize it here; for a successful result would generally, if not always, be thwarted by an infectious matter—the fluids of the mouth even—that might enter the root-canals subsequent to the first step in the treatment of the case. No matter how badly broken down the crown of the tooth may be, or swollen the surrounding parts—an alveolar abscess of the character just described will readily yield to the treatment above indicated—as a rule.

The treatment of the teeth anterior to the bicuspids is, in some respects, still more simple: the roots being straight, and the canals more cylindrical. In these teeth it is sometimes better, if not too much weakened thereby, to make a direct opening into the canal from the lingual aspect. In other respects, the treatment of these teeth is the same as in the case just described. It is hardly necessary for me to state that this treatment applies equally well to abscessed roots upon which artificial crowns are to be secured.

The treatment of fistulous abscesses, that are associated with the molar teeth, is somewhat more complicated, inasmuch as it is more difficult to obtain free access to the root-canals; but when these are once opened, a cure is generally quite easily effected by adopting, essentially, the same method as that described for the single-rooted tooth.

In the foregoing I have had in view those alveolar abscesses in which fistulous openings had been already established. We will now briefly consider the treatment of those abscesses which are generally far more difficult to manage. Having no fistulous outlets they are sometimes called "blind" abscesses; some of these yield readily to treatment especially if they are not of long standing, yet they require more cautious management. The cavities of decay and root-canals should be cleansed, as in the cases just cited, but greater care should be exercised in the manipulation, lest some of the debris be forced through the apical foramen. It will be readily seen that this care is more necessary in the treatment of the lower teeth than of the upper. After the operation of cleansing the cavity and root-canals is completed, if the pus does not already flow into the canal, a broach should be passed through the foramen and the pus and serum that follow the withdrawal of the instrument should be removed or wiped out until it ceases. Then make a piston by winding cotton around a suitable instrument—the head of a Gate's drill does very well—and saturate it with eucalyptus oil or phenol sodique. With this as much of the pus as possible should be forced or pumped from the abscess. Of course the cotton should be removed from the instrument and renewed many times. By this means the remedial agent will be forced through the foramen and drawn out again, bringing with it a mixture of purulent matter. By repeating this operation several times nearly all the contents of the sac will be removed. I prefer the eucalyptus oil or phenol sodique to creosote or carbolic acid in this operation, because the latter, by coagulating the albuminous ingredients of the pus, affect its fluidity, and thus increase the difficulty of drawing it through the apical foramen. After the contents of the sac are measurably exhausted, a little creosote may be injected into the abscess, and the root, or roots, be filled with a light pellet of cotton, and the cavity be sealed up. If there is not a sufficient quantity of creosote in the canals to saturate the cotton, a little should be added. On the second or third day subsequent to this treatment the temporary stopping, and the dressing in the canals, should be removed. To a practiced eye, the appearance of the dressing taken from the canals will often indicate the condition of the parts beyond the apex. If pus should be found in the meshes of the cotton, or if serous fluid, or pus in small quantities, should in a few minutes ooze through the apical foramen, or, if we find there has been a leakage of the temporary stopping, the same treatment should be repeated. The second treatment will, as a rule, be sufficient to cure the abscess. But, in most cases, I would advise the removal of the second dressing after about a week's time, and again to pack the root canals tightly with cotton moistened with creosote; and then to securely fill the remaining cavity with a trial filling that might remain for a month or more.

This is all the treatment that is required to cure the great majority of cases of this class. But occasionally we meet with a case that will not yield so readily. In such a case, after a faithful course of treatment with creosote and eucalyptus has proved ineffectual, it would be well to substitute aromatic sulphuric acid. The effect of this remedy in these stubborn cases, though somewhat painful, is often truly wonderful; and one application is frequently sufficient to arrest the secretion of pus. The subsequent treatment would be the same as that adopted in milder cases. In some of these cases—especially those of long standing—when the external wall of the abscess has become thin and yielding, the quickest, and perhaps the least painful, method to pursue, would be to produce an artificial fistula with a trephine, or any other instrument.

The lesions may be then treated as before described for fistulous abscesses. Of course, if there be necrosis of the osseous parts, the removal of the sequestrum, and the substitution of aromatic sulphuric acid, will be indicated, and will generally result in a speedy cure.

Abscesses, with fistula opening externally upon the face, yield readily to this treatment, without the loss of the teeth that are associated with them. Even those of many year's standing may often be cured by a single injection of creosote or carbolic acid. You are all aware that abscesses of this nature quite frequently arise from the death of the pulp in the inferior incisors that are entirely free from decay; the cause being frequently overlooked by the medical, and sometimes even by the dental practitioner. Cases of this kind, which had been treated externally for a series of years, have come under the observation of many of you. By applying the same principles recommended in the first case described, an abscess of this character may be cured almost invariably by an operation requiring thirty minutes', or at most an hour's time, and the tooth retained in its usefulness.

Perhaps it may not be amiss, here, for me to suggest that the opening should be made posterior to the cutting edge of the tooth, I speak of the inferior incisors, and in as direct a line as practicable with the longitudinal axis of the root canal. This is easily accomplished by the aid of the dental engine, and a right-angle attachment armed with a triangular spear-pointed drill, having a small and slightly flexible shank. I need not detain you with the further details of this operation, as they have been fully suggested by the foregoing treatment of the bicuspid. I will bring this imperfect sketch of the details in practice to a close after a few words concerning the treatment of incipient alveolar abscess. Of course it is often difficult to distinguish an incipient abscess from periostitis when the latter is occasioned by the mephitic gases or other irritating matters that find their way through the apical foramen. But this is of no practical importance, since the same treatment that is adopted for the one is applicable to the other. In cases of incipient abscess the rubber dam should be applied as in the cases before named, and after having removed the parts softened by decay, the dead pulp or other matter should be carefully removed from the canal. It would be better not to thoroughly remove the debris at the first sitting, lest in the attempt to do this we force matter through the foramen, and thus aggravate, rather than ameliorate, the trouble. After this partial cleansing of the cavity, a pledget of cotton saturated with eucalyptus, or dilute creosote should be delicately introduced into the canal and the cavity sealed up with cotton and sandarach varnish. In this operation pressure should be carefully avoided. The margins of the surrounding gums should then be touched with a solution of iodine in creosote. This may be conveniently done with a small spatula that will pass between the circumjacent gums and the neck of the tooth. After three to five days the cavity of decay and the root canals, should be thoroughly cleansed, and the latter now filled densely with cotton moistened with a little creosote, and the former with gutta-percha or some other impervious material. For an incipient abscess—periodontitis from the cause already named—thist reatment will, with very careful manipulation, generally prove all-sufficient. It is, of course, understood that the fluids of the mouth must be absolutely excluded from the cavity, from the commencement of the treatment until the cavity is permanently filled.

